# Editorial: Rising stars in infectious diseases—Surveillance, prevention and treatment: 2022

**DOI:** 10.3389/fmed.2023.1234922

**Published:** 2023-07-04

**Authors:** Senthil Kumaran Satyanarayanan, Zisis Kozlakidis

**Affiliations:** ^1^Mind-Body Interface Laboratory (MBI-Lab), Department of Psychiatry, China Medical University Hospital, Taichung, Taiwan; ^2^International Agency for Research on Cancer, World Health Organization, Lyon, France

**Keywords:** infectious diseases, surveillance, prevention, treatment, infectious disease pathogens

Contemporary science and medicine have made extensive strides in understanding, preventing, and controlling infectious diseases. However, the dynamics of pathogenic encounters and human responses continue to generate multifaceted puzzles for the global scientific community. In a world continuously challenged by the threat of infectious diseases, advancements in disease management and understanding have never been more critical. From the relentless struggle against COVID-19 and tuberculosis (TB) to the long-standing fight against human papillomavirus (HPV) and cytomegalovirus (CMV), the quest for effective prevention, diagnosis, and treatment strategies remains at the forefront of public health research. In this editorial, we discuss insights gained from six recent studies published under the Research Topic “*Rising stars in infectious diseases—Surveillance, prevention, and treatment*” that reflect the current state of infectious disease research.

The studies published within this Research Topic highlight the breadth and depth of ongoing research in the infectious disease field. The Research Topic does not focus on a singular condition or pathogen, but presents current developments across the field. Thus, each one of these studies provides important insights into various aspects of infectious diseases and public health, focusing on viral and bacterial pathogens. While the diseases studied in each publication are distinct, some common threads link them together, creating a cohesive flow between different diseases. These studies emphasize the importance of early intervention, understanding local epidemiology, and the complex interplay between infection, immune response, and disease progression. Furthermore, they highlight the need for ongoing surveillance and research to inform public health strategies and improve disease management. The studies published under the Research Topic are briefly presented in the following paragraphs.

First, Liu et al.'s quasi-experimental study investigated the effect of nasal irrigation on the duration of symptoms and nucleic acid conversion in adults infected with the Omicron variant of COVID-19. The authors utilize hypertonic saline nasal irrigation as an add-on to conventional treatment, contrasting this with a control group. Remarkably, they find that nasal irrigation, a simple and cost-effective intervention, appears to shorten the time of nucleic acid negative conversion, a proxy for viral clearance, with no significant improvements in symptom disappearance time. This research opens a new door for at-scale, non-pharmacological intervention that may have broad implications, especially in resource-restricted settings.

While some battles against infectious diseases seem to progress, the war against TB in China appears to be hampered by a formidable challenge: loss to follow-up (LTFU) (Jiang et al.). Jiang et al. report that patients' treatment histories, clinical characteristics, and socioeconomic factors play pivotal roles in predicting LTFU. The authors suggest that early assessment and intervention after diagnosis can improve patient engagement and treatment adherence, improving health outcomes and eventually disease control. This study serves as a stark reminder that social and systemic factors are integral to infectious disease control in addition to biotechnological advances.

Complementing the investigation of TB management is a study by Luo et al. analyzing relative monocyte levels as prognostic biomarkers in TB patients with anemia. The researchers provide insightful observations on the potential role of monocytes in predicting disease progression and outcomes. Elevated monocyte levels were associated with poor prognosis and poor cavity pulmonary closure in TB patients with anemia. This research sheds light on how immunological parameters can inform patient management and prognosis, adding to the existing web of TB control strategies.

At the forefront of disease transmission analysis is a study by Lin et al. which employs conventional epidemiological survey data and whole-genome sequencing to elucidate the genotypic distribution and transmission characteristics of *Mycobacterium tuberculosis* in schools in Guangzhou, an area with one of the highest TB incidences in China. The results suggest that the spread of TB cases in Guangzhou schools mainly originates from community transmission, emphasizing the need for stronger surveillance and containment measures to halt TB spread in educational institutions.

These three studies focus on critical aspects such as factors influencing treatment adherence, monocyte levels as prognosis biomarkers, and TB transmission in schools, respectively. All three studies on TB adopt an epidemiological perspective, analyzing patient data retrospectively to uncover trends and insights. These studies are also set in China, indicating the relevance of TB as a public health issue in the country. Each study considers various patient-centric variables such as past medical history, socioeconomic factors, immune response, and environment (schools). Most importantly, these studies collectively contribute valuable insights into understanding, predicting, and managing TB, thereby informing public health strategies and patient care protocols.

Shifting focus from TB to another infectious disease with a global impact, we delve into a retrospective study on the prevalence and genotype distribution of high-risk HPV among women in Liaocheng, Shandong Province, China (Zheng et al.). Zheng et al. highlight the significance of understanding the regional HPV prevalence and genotype distribution to optimize vaccination strategies. The finding that HPV16, HPV52, HPV58, and HPV53 are widely distributed underscores the urgency of strengthening preventive measures, particularly vaccination, to curb the high HPV infection rates in this region of China.

Finally, Ye et al. provide an overview of cytomegalovirus-associated anterior uveitis and glaucoma. This disease, primarily affecting middle-aged men, underscores the diversity of human–pathogen interactions, which may lead to varying clinical manifestations and complications, such as secondary glaucoma. The authors highlight the importance of diagnostic tools and combination therapies in managing this condition, again underlining infectious diseases' complexities and multifaceted clinical natures.

Despite current advances for COVID-19, the publications in this Research Topic serve as a reminder that much more work is required to understand the many infectious diseases that currently threaten public health. From simple non-pharmacological interventions for COVID-19 to the multifaceted challenges of TB, HPV, and CMV control, the fight against infectious diseases necessitates an integrated approach. By unraveling the intricacies of these diseases, we can pave the way for better public health outcomes, illuminating the path toward a healthier global community.

## Author contributions

SS and ZK wrote the first draft of the manuscript. The final manuscript has been reviewed by all authors and is approved by them.

